# Caring for Patients Anywhere, Anyhow: How Emergency Department Crowding and Boarding Shapes Professional Identity in Emergency Medicine Training

**DOI:** 10.5811/westjem.64726

**Published:** 2026-11-02

**Authors:** Marlena Skrabak, Ezra Bisom-Rapp, Esther H. Chen, Christopher R. Peabody

**Affiliations:** * University of California, San Francisco, School of Medicine, San Francisco, California; † University of California, San Francisco, Zuckerberg San Francisco General Hospital, Department of Emergency Medicine, San Francisco, California

## Abstract

**Introduction:**

Emergency department (ED) boarding is a well-documented crisis associated with adverse patient outcomes, healthcare worker burnout, and moral injury. Although prior work has documented operational and educational impacts, including reductions in resident productivity and procedural exposure, less is known about how sustained boarding shapes professional identity formation. We sought to explore how sustained ED boarding shapes the learning experiences and professional identity formation of emergency medicine (EM) residents, as perceived by both residents and the attending physicians who train them.

**Methods:**

Using a constructivist paradigm, we conducted a qualitative study using semi-structured interviews with residents and attending physicians from a 4-year EM residency program with rotations across 5 clinical sites, between July and August 2025. Two researchers independently coded transcripts and iteratively refined a shared codebook, continuing until additional interviews were unlikely to add new insight, guided by the concept of information power. We used reflexive thematic analysis, treating researchers’ clinical and analytic judgment as a resource for identifying patterns of meaning, and prioritized participants’ own construction of meaning over the frequency of any single idea, consistent with an interpretive phenomenological approach.

**Results:**

We interviewed 13 emergency physicians: 6 residents and 7 attending physicians (range: 1–30+ years after residency). Three overarching themes emerged: (1) Educational challenges and opportunities—boarding fragments diagnostic ownership, narrows the acuity spectrum, and limits authentic undifferentiated encounters, although attending physicians adapt through structured hypotheticals and micro-teaching); (2) Shifts in the clinical environment—operational and structural adaptations create educational dead space, constrain the therapeutic alliance, and generate role ambiguity around boarded patients); and (3) Moral strain and suffering—moral injury and combined suffering is not peripheral to training but embedded within it, shaping how residents interpret professional responsibility and integrity. The three themes exist in a triangular relationship, with suffering at the foundation.

**Conclusion:**

Boarding appeared to function as a formative context for professional identity formation, not merely a disruptive one. Residents described learning systems-level practice, advocacy, and emotional regulation, often through loss and moral injury, alongside attending physicians navigating the same. These findings suggest that recognizing boarding as a structural condition of training may reorient the responsibility of graduate medical education: formalizing adaptive teaching strategies, creating structured opportunities to process moral strain, and committing to advocacy that treats boarding as the educational crisis it is.

## INTRODUCTION

Boarding of admitted patients in an emergency department (ED) remains one of the most pressing operational health system challenges.^1^ Boarding is associated with adverse patient outcomes, including increased mortality, higher readmission rates, prolonged hospital length of stay, decreased patient satisfaction, and increased healthcare costs.^2^–^5^ Further, ED boarding contributes to healthcare worker burnout, workplace violence, and moral injury, defined as distress stemming from knowing what care should look like but being unable to provide it.^6^–^8^

Less is known about the educational impact of boarding on emergency medicine (EM) residency training.^9^,^10^ Some studies show a negative impact on EM training, including lower resident productivity (ie, patients seen per hour), less exposure to undifferentiated patients when workups are initiated in triage, limited exposure to critically ill patients who might be diverted elsewhere, and decreased physician wellbeing.^11^–^13^ Other studies show that boarding has limited impact on EM training as measured by number of procedures performed or potential impact on EM milestones.^14^,^15^ Moreover, faculty adapt their approach to bedside teaching, such as by changing teaching locations or modifying supervision patterns.^6^,^11^,^16^,^17^ Others looked at crowding’s effect on the Accreditation Council for Graduate Medical Education (ACGME) core competencies.^18^ However, residents’ or attending physicians’ perspectives regarding the effect of significant ED boarding on achieving these milestones remains unknown.^13^,^19^,^20^ Building on this foundation, we sought to explore how sustained ED boarding conditions shape an EM trainee’s professional identity formation.

Professional identity formation theory describes the process by which socialization, experiential learning, and the learning environment together forge a person’s sense of self and role.^21^ Rather than being acquired as a discrete skill or body of knowledge, professional identity develops iteratively through repeated participation in the authentic work of a profession, shaped not only by formal teaching but by what is modeled and normalized within an environment.^22^ Under this framework, the conditions in which trainees practice are not incidental to their training but constitutive of it. By gaining a deeper understanding of how boarding may fundamentally change the clinical experience of EM residents, this study sought to inform programmatic strategies to support residents in becoming independent practitioners who can care for patients in complex clinical environments.

## METHODS

### Study Design and Setting

We conducted a series of individual interviews with faculty and residents from a 4-year EM residency training program with clinical rotations in multiple practice settings: a quaternary academic medical center; an urban safety-net hospital and Level I trauma center; a quaternary children’s hospital; a Veterans Affairs medical center; and a safety-net children’s hospital and Level I trauma center. Residents were eligible to participate if they had rotated in at least two different clinical sites. Our organization’s institutional review board determined this study was exempt (#23-38734). This study is reported in accordance with the Standards for Reporting Qualitative Research.^23^

**Population Health Research Capsule**

What do we already know about this issue?

*Emergency department (ED) boarding is linked to worse patient outcomes, burnout, and moral injury; its educational effects on emergency medicine (EM) residents remains poorly understood.*

What was the research question?

*How does sustained ED boarding shape the professional identity formation of EM residents?*

What was the major finding of the study?

*Boarding shaped EM residents’ professional identity through interconnected educational and environmental changes, with suffering as the foundational theme.*

How does this improve population health?

*Recognizing boarding as an educational crisis may guide training reforms that prepare physicians to provide care and advocate for patients under systemic strain.*

### Study Participants

Author CP recruited participants using the department’s email listserv, reaching 152 attending physicians, fellows and residents, in June 2025 over a 3-week period to complete a 30-minute interview. Author MS scheduled and conducted all the interviews and continued until additional interviews were unlikely to add additional insights, such that, per the concept of information power, saturation depended on the specificity of the sample, the depth of dialogue, and the analytic approach, rather than a fixed number.^24^,^25^ All participants provided verbal consent to participate in the study. Each interview lasted approximately 30 minutes and was conducted between July and August 2025. Participants received a $25 gift card for their time.

### Interview Guide Development

Authors MS, EB, CP, and EC developed two interview guides, one for resident physicians and another for attending physicians. Interview guides focused on clinical education and skill development related to ACGME milestones, exploring perspectives on what it means to learn and teach in boarding environments.^26^ Questions addressed adaptations, teaching practices, and values-based work to elicit how the boarding crisis shapes the experience of becoming an emergency physician and the experience of teaching future emergency physicians (see Appendix).

MS, who conducted all interviews, had no supervisory, evaluative, or authoritative role over the people interviewed. Interviews were conducted, recorded, and transcribed using HIPAA-compliant Zoom videoconferencing software version 7.0.6 (Zoom Communications, Inc). MS de-identified all interview transcripts, and MS and EB reviewed the transcripts for accuracy.

### Analysis

MS and EB coded the interview transcripts using ATLAS.ti (Scientific Software Development GmgH), a qualitative data analysis software to organize, label (ie, code) and compare text excerpts across interviews. We used reflexive thematic analysis—identifying and developing patterns of meaning (ie, themes)—because it treats researchers’ clinical and disciplinary knowledge as an analytic resource rather than a source of bias to be minimized.^27^ MS and EB were medical students with an interest in EM who had rotated at the study sites without prior personal relationships with participants, while CP and EC were EM faculty whose clinical and educational experience informed the interpretive process. To develop a preliminary codebook, MS and EB collaboratively reviewed and discussed one transcript, generating descriptive codes grounded in participants’ language, then iteratively refined the codebook throughout the analytical process, reviewing each other’s codes and reconciling discrepancies to full consensus, with CP resolving disagreements. CP and EC independently reviewed the codes and met multiple times with MS and EB to discuss thematic patterns, synthesize codes into higher-order themes, and examine their relationships. Furthermore, our analytic approach was informed by interpretive phenomenological analysis, which is concerned with how individuals make sense of their lived experience.^28^ This approach prioritizes participants’ construction of meaning over the frequency with which any idea occurred to develop a nuanced narrative of how their experiences learning and teaching in a boarded ED.^29^

## RESULTS

We interviewed 13 emergency physicians: 6 residents and 7 attending physicians with varying years of practice. All residents had completed rotations at multiple ED sites. No postgraduate year one residents participated because they did not have time to rotate through multiple ED sites during the study period. Attending physicians’ primary clinical work sites were the quaternary academic medical center or urban safety-net hospital. Additional demographic information is included in Table 1.

Participants described boarding as fundamentally restructuring the experience of EM graduate medical education. Boarding alters what residents are able to learn, how they learn it, and how they come to understand their role as emergency physicians. Our analysis yielded three overarching themes: (1) educational challenges and opportunities; (2) physical and conceptual shifts in the clinical environment; and (3) moral strain and suffering (see Table 2).

Changes to clinical workflow reshape educational exposure, environmental restructuring creates new gaps and unexpected opportunities, and repeated exposure to constrained care shapes how residents interpret their professional responsibilities and values. The three higher-order themes, developed through reflective thematic analysis and interpreted through interpretive phenomenological analysis, exist in a triangular relationship. Suffering recurred within participants’ accounts of education and the environment, and the two latter themes were deeply intertwined. Therefore, to represent thematic relationships accurately, suffering belonged at the base as a foundational, underlying theme rather than a parallel one, with the two other themes in a reciprocal, rather than hierarchical, relationship (see Figure).

### Theme 1: Educational Challenges and Opportunities

Boarding changes not only the volume of patients that residents see but the type of ownership, reasoning, and decision-making they practice. Educational challenges and opportunities emerge directly from boarding’s restructuring of clinical work.

#### 1.1 Learning in a constrained clinical system

Residents frequently assume care of patients whose workup has already been initiated, or whose diagnostic trajectories have largely been determined. They inherit partially completed plans:

…then you get signed out all these patients that are pending workups, and […] you’re following someone else’s plan, who’s kind of like giving you instructions on what to do. (Resident #2)

Residents describe fewer opportunities to construct differential diagnoses and more time managing throughput. In response, some residents describe how they intentionally sought lower-acuity patients they believed they could discharge efficiently:

I’ll pick up people that I’m like, oh, I could discharge you with some Tylenol […] I’m almost looking for those low-hanging fruit, because this patient that I know needs to be admitted is going [to] sit in the waiting room, and they’re not going to get the medicine they need. (Resident #5)

Additionally, residents describe wanting to see more patients but are limited by system capacity. They identify constraints on not only educational opportunity but also the development of clinical confidence and autonomy.

#### 1.2 Adaptive teaching strategies within constraint

Attending physicians describe deliberate attempts to reconstruct educational value within constrained conditions. One common strategy is the use of structured hypotheticals that function as compensatory tools to preserve clinical reasoning when authentic undifferentiated encounters are limited:

Okay, we didn’t get to do this before we found the diagnosis for this patient. Let’s talk about what we would have thought about and why we would have gone down this route. […] What you can’t do is let the resident evaluate a patient without knowing what they already have. (Attending #6)

These scenarios also become opportunities to teach residents how to reimagine a system that prioritizes patient-centered care: “What would we prioritize if we didn’t have the limitations brought by boarding?” (Attending #6). A reliance on hypotheticals reframes and builds creative solutions. Further, attending physicians, through micro-teaching moments, share interesting cases across teams, teach during and after sign-out, and message residents about patient outcomes left unfinished during shifts. What is being transmitted extends beyond clinical reasoning:

The learners are not getting the medical knowledge […] if they had the patient on their own. But they are learning about systems-level practice and also about internal management of emotions and processing of emotions, which is an important skill too. (Attending #3)

These strategies mitigate educational loss, but residents note how the burden of curricular preservation is dependent on each attending and their bandwidth rather than standardized curricular components.

#### 1.3 Communication, ownership, and systems-based practice

Residents describe learning how to articulate system constraints while maintaining professionalism:

One of our core requirements in EM training is the ability to communicate with consultants, other services and admitting teams. And something I’ve taken away is, how do I translate my strong emotions around boarding […] into something productive [for patients]? (Resident #4)

Boarding heightens residents’ communication skills, as they learn to describe the effects of boarding to teams not on the frontlines, in hopes of improving patient care and flow. Communication with patients is conversely impacted. The lack of privacy and space compromise the therapeutic alliance, which harms not only patient care but also how trainees feel they learn during and from patient encounters:

…when we don’t have a good rapport with our patients, then learning in that relationship really takes a hit. (Resident #2)

Residents also experience role ambiguity when it is unclear which interdisciplinary team—inpatient or ED—is caring for patients boarded in the ED. The “limbo of responsibility” (Resident #3) complicates the ownership of patient care and requires residents to negotiate boundaries in real time, intensifying systems-based learning, often in ways that are reactive rather than structured.

### Theme 2: Shifts in the Clinical Environment

Boarding restructures not only what residents learn, but the physical and conceptual environment in which they learn. Participants describe how the environment of the ED (its operational flow, spatial constraints, and structural adaptations) creates conditions that also reshape clinical exposure and resident identity.

#### 2.1 Operational flow and structural adaptations

When evaluating the environment of the ED, the flow of patients through the system is a metric of capacity, and capacity determines when residents are learning and who they are learning from. When only the highest-acuity patients are brought to monitored beds due to reduced ED capacity, residents’ exposure to the full acuity spectrum narrows:

Part of the education for residents is to prepare them to work up and manage a wide variety of diagnoses and chief complaints. By limiting what comes to the back, you truncate what they’re exposed to. (Attending #5)

Consequently, patients with noncritical presentations are seen in waiting rooms, hallways, or triage cubicles without the same privacy or monitoring as ED beds. The spatial fragmentation of care limits the quality and consistency of clinical learning.

Furthermore, structural adaptations, such as nurse practitioners or attending physicians in triage, expedite discharge pathways and alter room allocation patterns.

The residents are never working in the triage area. That’s just not part of their workflow ever. And yet, rapid assessment, interview, and evaluation as a first point of contact is a really important skill to learn in emergency medicine. (Attending #3)

Attending physicians in this study noted that these structural responses, while addressing throughput, create educational dead space in which residents are absent from key components of emergency care. And they recognize that these environmental shifts alter the informal curriculum of residency.

#### 2.2 Capacity, constraint, and the interrupted feedback loop

Attending physicians described clinical exposure and the ability to manage high patient volumes as critical components of becoming a competent emergency physician, and they contrast this with how boarding affects a physician’s ability to manage a high load:

I find, when we are open, [the residents] don’t own all the patients. They’re not picking them all up, they’re not seeing them all, they’re overwhelmed. […] They’re capable of it—they just haven’t had to flex that muscle. (Attending #3)

While the resident narratives echo this feeling, they also express feeling limited by the stagnated capacity of a boarding system:

I’ve had the bandwidth to see more patients, or to do more learning essentially. The department doesn’t have the capacity for me to do that. And I feel like that really shouldn’t be the case. (Resident #5)

Attending physicians and residents describe a mismatch between a resident’s capacity and the system’s capacity to use it. Several attending physicians note how residents may not learn where their ceiling is and instead calibrate their ability to an over-filled system.

#### 2.3 Role diffusion in a conceptual environment

Boarding not only reshapes the physical environment of the ED but also reshapes residents’ roles and sense of responsibility within the system. Boarded patients have inpatient teams nominally managing their care, yet they remain in the emergency space. Residents describe uncertainty about who is responsible for a boarding patient, particularly if an ED nurse calls them about a boarded patient care issue:

Who’s taking care of [boarded patients]? And are they trained to do that? And how do you handle task-switching between, “This is a patient who should have been in the ICU 6 hours ago and is getting maximum BiPAP therapy for severe asthma…,” and then over here is a new patient that I need to start from scratch. (Resident #3)

Residents are also learning to redefine what their role in a challenging system looks like. Benefits of this environmental shift were also noted: One resident reflected that increased exposure to boarded inpatients made them more fluent in inpatient communication and better able to articulate clinical reasoning to consulting teams. Furthermore, attending physicians described that figuring out how to acknowledge environmental changes is itself a part of their patient-care workflow and therefore the workflow they model for residents:

How do you have that kind of a conversation with patients [about the broken system] without disparaging your leadership, disparaging the hospital, disparaging the system—because that leaves the patient with significant mistrust? (Attending #3)

Physicians find themselves acting as mediators between a malfunctioning system and the care their patients need.

### Theme 3: Moral Strain and Suffering

Beyond educational and environmental shifts, participants describe how boarding shapes the emotional conditions under which professional identity formation occurs. While learning clinical skills, residents also learn what it means to practice in a system that routinely constrains care. The theme of suffering permeated each interview—the suffering of patients, the suffering of peers, their own suffering, and the system suffering.

#### 3.1 Shared moral injury as part of training

Resident and attending physicians illustrate morally injurious cases:

I had a patient who had a broken pelvis. I cared for them entirely in the waiting room. […] At the end of the day, the patient said, ‘Thank you so much. This place is wonderful.’ I had to stop them there for a second and say, ‘You never left the waiting room, and you’re telling me this was a great experience?’ […] So despite me feeling really ashamed that they were being cared for this way, some patients still have the perception that they’re getting good care. (Attending #4)

These experiences are visible to learners. Attending physicians’ moral strain (and shame) becomes part of the emotional curriculum residents witness and absorb.

Residents similarly describe situations in which resource scarcity limits their ability to act:

I have a really sick asthma patient, and I don’t have a room to put them in, and I don’t have a nurse to administer medications to treat them. These start to become problems that I have less and less control over. (Resident #4)

Training occurs within repeated exposure to constrained care, shaping how residents interpret responsibility, agency, and professional standards.

#### 3.2 Distrust and identity tension

Residents describe tension between their expectations of practicing at a quaternary care center and the reality of delayed care. They worry about eroding patient trust and their own sense of integrity:

And we’re just telling [patients in pain] to sit in this chair for however many hours [until] we think we could get a room. I feel like I add to the distrust of these people… (Resident #2)

Boarding introduces identity tension: residents learn to practice emergency medicine while simultaneously confronting conditions that contradict their ideals of what emergency care should be and how it should be provided. They lose “curiosity or a fresh perspective because a lot of the work has already been done… [and] feel more like a cog in a wheel” (Attending #3). And they see their attending physicians experience the same.

#### 3.3 Modeling integrity under constraint

Attending physicians describe explicitly naming injustice with both patients and learners:

‘Look, I’m really sad that this is what I have to tell you. I’m really angry that this is the way that this has to be right now. This is why I can’t put you in a room, and I have to discharge you. And I agree it’s not fair.’ (Attending #3)

Such modeling reframes emotional acknowledgment as professional practice rather than weakness. Residents observe how faculty maintain integrity while adapting workflow to mitigate harm. Coping strategies, emotional processing, and advocacy become part of boarding’s hidden curriculum:

We call [boarding] part of the “hidden curriculum.” …while we shouldn’t validate the healthcare system in the US by saying boarding is okay…we’d say, *I can become a better emergency medicine doctor by caring for these patients for longer*. (Resident #4)

#### 3.4 Learning medicine through suffering

Residents describe shifts as if being in “survival mode”:

You go into your shift thinking, “I’m just going to survive.” The focus isn’t on learning; it’s on getting through the shift without crying and making sure nobody codes in the waiting room. (Resident #1)

Sustained exposure to suffering—of patients, of peers, of teachers, of themselves—affects motivation and engagement. Attending physicians in this study acknowledge how emotional processing is an essential component of emergency medicine. Yet learning under persistent moral strain requires residents to integrate ideals of patient-centered care with the realities of systemic constraint. Participants questioned what is being transmitted: resilience, adaptability, normalization of dysfunction, desensitization:

I don’t know that anyone can really teach to this non-ideal scenario—but what they’re doing is just trying to adapt and encouraging us to adapt with it, to the extent that’s possible” (Resident #1)

“I think [boarding] is pretty demoralizing a lot of days because you’re like, *I’m not doing anything*. (Resident #6)

## DISCUSSION

To our knowledge, this is the first study to examine the relationship between ED boarding and a trainee’s professional identity formation as an emergency physician. This study reframes ED boarding as more than an operational stressor or educational inconvenience but rather as a structural condition of training that shapes how residents interpret clinical responsibility, system constraints, and professional integrity. The moral distress of learning to care for patients under boarding conditions was echoed in every interview. Prior research has linked boarding to clinical burnout and stress.^12^ Our findings suggest that moral strain is embedded within current emergency medical education. Professional identity formation theory emphasizes that identity develops through social participation in an authentic space.^21^

Each of the three themes maps onto a core mechanism of professional identity formation: Theme 1 reflects socialization through altered clinical participation; theme 2 reflects how the learning environment itself is restructured; and theme 3 reflects values-based, relational work of internalizing a professional identity under strain. In our study, that authentic space is characterized by repeated ethical tension—moral strain (theme 3) infiltrates and feeds into education and the practice environment (themes 1 and 2). Residents’ thinking about diagnostics is shaped by their experiences of fragmented diagnostic ownership. It colors how they interpret the physical and conceptual constraints of the ED. It impacts their relationship with patients and other clinicians. Moreover, our data suggest that boarding can lead to resignation and desensitization. Theme 3 explores how this process is linked to the presence of and residents’ reactions to suffering, either collectively (when an attending chooses to model integrity and name injustice openly) or individually (when a resident enters a shift oriented toward survival).

The data highlight how the documented structural shifts of boarding (reduction of productivity and altered supervision/exposure patterns) are internalized by trainees. When diagnostic ownership is fragmented, when triage decisions are displaced, when patient flow is constrained, residents are not just practicing less, they are practicing differently. Similar adaptations in bedside teaching are described by faculty, such as the use of structured hypotheticals and micro-teaching within workflows, mirror principles found in simulation and case-based learning. During the COVID-19 pandemic, EM programs reported similar compensatory strategies to maintain competency development amid reduced clinical exposure.^30^ In our setting, these adaptations have emerged organically and variably, rather than through deliberate curricular reform. As boarding persists, reliance on informal faculty innovation may produce uneven educational experiences unless these strategies are formalized. Furthermore, if simulation and hypotheticals as modes of learning procedural skills supplement resident education, long-term retention will need to be studied.^31^,^32^

Our learners also highlight the hidden curriculum of learning emergency care in a boarded ED. Research describes how ED wait times socialize trainees into norms of coping with dysfunction.^33^ Similarly, boarding appears to communicate implicit lessons about what constitutes acceptable care, how to manage institutional limitations, and how to reconcile ideals with practice. Without explicit reflection, prolonged exposure to constrained care environments may normalize clinical compromise. When faculty name injustice and model ethical discomfort, they can interrupt that normalization and convert moral tension into reflective teaching.

These findings suggest several implications for graduate medical education. First, operational decisions should be evaluated for educational impact, particularly when workflow restructuring alters diagnostic ownership.^34^ Because education remains a key mission of academic EDs, education leaders could collaborate with operational leaders to assess the educational impact of operational decisions. Second, compensatory teaching strategies, such as hypothetical-based reasoning exercises, may require formal integration to ensure consistency across learners. Programs may help residents set their individual learning goals by providing dashboards with aggregate data about diversity of cases and procedure logs so that they may work with their supervising faculty to mitigate their knowledge gaps. Third, programs may benefit from creating structured opportunities to address moral strain directly and preventing it from functioning solely as a hidden curriculum.^35^ Residents and faculty may share experiences and support one another through restorative justice community circles, case discussions, and narrative medicine reflective exercises.^36^–^39^ Finally, if boarding reflects a durable feature of contemporary emergency care, residency training must explicitly define which competencies require protection (such as diagnostic reasoning from undifferentiated presentations) and which new competencies should be deliberately cultivated (such as systems-based advocacy), while tracking progression through these milestones to update each resident’s learning plan.

## LIMITATIONS

This study reflects the perspectives of 13 physicians and was conducted at a single academic site with a specific configuration of training sites and boarding patterns. Findings are not intended to be statistically generalizable and should be interpreted as context-specific, analytic insights.^40^ Participants self-selected, which may introduce bias toward those with strong views or experiences regarding boarding; however, this self-selection may also have allowed us to capture the voices of those most affected and most reflective about the impact of boarding. Future multisite studies would help establish the transferability of these themes across institutions with differing boarding severity, resource constraints, and training structures.

## CONCLUSION

Boarding appeared to function as a powerful context for professional identity formation, shaping how residents develop core competencies in emergency care in a constrained clinical environment. These lessons are learned within the undercurrent of moral distress and co-constructed with faculty who can intentionally fill in knowledge gaps and model how to practice within these realities. These findings suggest that recognizing boarding as an educational crisis rather than simply an operational may warrant intentional curricular support and institutional advocacy to sustain trainees, educators, and patients, and merits testing across broader training contexts.

## Supplementary Information



## Figures and Tables

**Figure:**
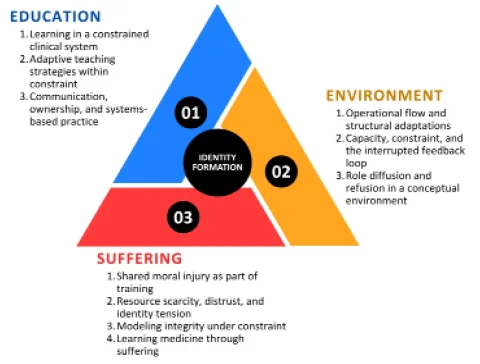
Triangular relationship among three themes emergent from interviews with 13 emergency physicians, in a qualitative study of the impact of emergency department boarding on graduate medical education and professional identity formation.

**Table 1:** Demographic characteristics of 13 emergency physicians (6 residents and 7 attending physicians) interviewed in a qualitative study of the impact of emergency department boarding on graduate medical education and professional identity formation.

	Resident physicians (*n* = 6)	Attending physicians (*n* = 7)
Sex†		
Man	2	3
Woman	4	4
Year in training (resident physicians)		
PGY 2	1	
PGY 3	2	
PGY 4	1	
Fellow	2	
Years in practice following residency (attending physicians)		
1–4 yrs		1
5–9 yrs		1
10–19 yrs		1
20–29 yrs		3
≥30 yrs		1
Sites of practice*		
Quaternary care hospital	6	2
Quaternary care pediatric hospital	5	
Urban safety net hospital	5	4
Urban pediatric safety net hospital	2	
Urban safety net hospital + Urban pediatric safety net hospital		1
Veterans’ Affairs hospital	4	

† Sex was self-identified by participants.

* For resident physicians, counts indicate the number of individuals who have completed rotations at each site, out of n = 6 participants.

*PGY*, post-graduate year; *yrs*, years.

**Table 2:** Overarching themes and representative quotes identified through reflexive thematic analysis of interviews with 13 emergency physicians, in a qualitative study of the impact of emergency department boarding on graduate medical education and professional identity formation.

Theme 1: Educational challenges and opportunities	
*Learning in a constrained clinical system*	“…then you get signed out all these patients that are pending workups, and […] you’re following someone else’s plan, who’s kind of like giving you instructions on what to do…” (Resident #2) “I’ll pick up people that I’m like, oh, I could discharge you…” (Resident #5)
*Adaptive teaching strategies within constraint*	“‘Okay, we didn’t get to do this before we found the diagnosis… Let’s talk about what we would have thought about…’ What you can’t do is let the resident evaluate a patient without knowing what they already have.” (Attending #6) “…if I see somebody who’s really interesting… I’ll try to share that information with the other residents. Even if that’s a patient I’m seeing in triage.” (Attending #2)
*Communication, ownership, and systems-based practice*	“…how do I translate my really strong emotions around boarding… into something productive? … ‘I have 10 patients with my name on them, but I also have 70 in the waiting room…’” (Resident #4) “…when we don’t have a good rapport with our patients, that part of learning… really takes a hit.” (Resident #2) “The positive takeaway… is that I’m much more familiar with inpatient care… I can articulate more clearly why a patient needs to be admitted.” (Resident #3)

**Theme 2: Shifts in the clinical environment**	

*Operational flow and structural adaptations*	“Part of the education for residents is to prepare them to work up and manage a wide variety of diagnoses and chief complaints. By limiting what comes to the back, you truncate what they’re exposed to.” (Attending #5) “…almost everyone else, who has anything from just less than a true emergency […] gets seen in the waiting room, or in a hallway, or in a little cubicle off to the side of the triage and vitals area.” (Resident #3) “The residents are never working in the triage area… And yet, rapid assessment… is a really important skill to learn in emergency medicine.” (Attending #3)
*Capacity, constraint, and the interrupted feedback loop*	“Those patients never get back to the ED and are never seen by the residents… they’ve mentioned feeling deficient in urgent care.” (Attending #9) “I’ve had the bandwidth to see more patients… The department doesn’t have the capacity for me to do that.” (Resident #5) “If you’re not seeing the breadth of clinical presentations… you’re missing out on learning about a huge swath of patients…” (Attending #5)
*Role diffusion in a conceptual environment*	“Who’s taking care of [the boarders]? And are the trained to do that? …” (Resident #3) “The positive takeaway from my residency experience in a boarding environment is that I’m much more familiar with inpatient care. Because of that, I can better advocate for my patients when they need admission.” (Resident #4)
**Theme 3: Moral strain and suffering**	
*Shared moral injury as part of training*	“[A patient with a broken pelvis] got CTs, an ortho consult, and all this stuff—and never left the waiting room. At the end of the day, the patient said, “Thank you so much. This place is wonderful.” I had to stop them there for a second and say, “You never left the waiting room, and you’re telling me this was a great experience?” […] That person didn’t deserve that.” (Attending #4) “These start to become problems that I have less and less control over.” (Resident #4)
*Distrust and identity tension*	“I hate not being able to do anything for [complex patients who are suffering] in the waiting room… [Patients who should receive thoracenteses] have to sit there, not being able to breathe, for longer.” (Resident #1) “I feel like I add to the distrust of these people…” (Resident #2)
*Modeling integrity under constraint*	I’d stepped over [a patient] numerous times to see other people that were ahead of him. […] he clearly needs to be admitted to the hospital, [but he] was going to have to go lie back out on the floor until we could find a bed for him [after triage]. And I said no, “I’m keeping him in the triage room on this gurney.” (Attending #3) “This is not right… What we’re going to do to mitigate the potential harm on our patient but still care for them within the system is to do this thing… […] when I leave, I know that I have been practicing with integrity.” (Attending #3)
*Learning medicine through suffering*	“[The learners are] not getting the medical knowledge teaching or learning that they would have gotten if they had the patient on their own. But they are learning […] about internal management of emotions and processing of emotions” (Attending #3) “The focus isn’t on learning, it’s on getting through the shift without crying…” (Resident #1) “…to keep putting yourself out there and keep seeing the patients… then you’re also just feeling so down and burnt out…” (Resident #2)

*CT*, computed tomography.
