# Supplementary material for: Caring for Patients Anywhere, Anyhow: How Emergency Department Crowding and Boarding Shapes Professional Identity in Emergency Medicine Training
Source: West J Emerg Med. 2026 Nov 2;27(6):1560–9. doi: 10.5811/westjem.64726 (PMC13762036; doi:10.5811/westjem.64726)
Supplement: Supplementary file 1 [file wjem-27-1560-s001.docx]

Appendix: Interview Guides for Attendings and Residents

**EMERGENCY DEPARTMENT BOARDING PROJECT**

Interview Guide for Attendings

**SECTIONS:**
Introduction (4)
Clinical Education & Skill Development (5)
Adaptations & Teaching Practices (4)
Closing & Reflections (4)

[Complete verbal consent script and obtain verbal consent before proceeding.]

[Acknowledge relevant demographic information already collected in pre-interview communications.]

**Research Question**
*How has emergency department boarding reshaped clinical education, bedside teaching, and skill development in Graduate Medical Education for Emergency Medicine, and how have residents and attending physicians adapted to the challenges and opportunities posed by the boarding crisis?*

**INTRODUCTION: ED**
Thank you for participating in this interview. This study seeks to understand how emergency department boarding impacts resident education, patient care, and physician well-being. Your insights will help us evaluate current conditions in the Emergency Department and identify both challenges caused by ED boarding and potential improvements to ED workflows and resident education.

**0.1** Can you describe to me what a typical shift of an emergency physician looks like?

**0.2** Overall, how would you characterize the learning environment for residents across the UCSF sites you’ve worked in?

**0.3** As you may know, boarding refers to holding patients in the ED after they have been admitted or transferred to the hospital but are unable to be moved to an inpatient bed or other designated area due to lack of availability. At which sites have you noticed this occurring, if any? How did working in under these conditions feel? (Do you think this problem is unique to…?)

**0.4** Think of a time when you were caring for a patient during a time of ED boarding. What was this experience like for you?

**SECTION 1: Clinical Education & Skill Development**
Next, I’d like to pivot to the impact of boarding on residency education. I am going to show you a graph representing the number of people boarded in the ED, as a reminder of when boarding may have been worse. [Share screen to present a chart of ED boarding levels by month].

**1.1** From your perspective as an educator and after reflecting on this graph or your last experience with boarding in the ED, how might you describe the impact of boarding on resident education?
*Probe*: Have you changed the way you teach at the bedside or supervise procedures? Can you give me an example of what this has looked like on a shift? What patterns have you noticed in learner exposure to critical cases or procedural volume?

**1.2** The ACGME has developed milestones that residents are evaluated on biannually to track their progress and skill development.

[Review list of 6 milestone buckets with participant: patient care, medical knowledge, systems-based practice, practice-based learning and improvement, professionalism, interpersonal communication.]

- Think back to a time when more patients were boarded than usual. How do you feel like resident progress on meeting these milestones was impacted during this time?
- During this time, how effectively are you able to assess resident competency or progression through these milestones?
  - *Probe*: Do you think the additional tasks required during times of boarding (when light or heavy) impede or facilitate opportunities for you, as a supervisor, to evaluate resident progress with these milestones and observe their growth? How does this impact your ability to give feedback on resident performance?
- Which milestones are most affected?

**1.3** How would you describe the impact of boarding on resident preparedness for independent practice?
*Probe*: Are there gaps that concern you? Does working with boarded patients facilitate the development of unexpected or unique strengths due to these conditions? In other words, do boarded patients provide new or unique opportunities for teaching that wouldn’t otherwise be available?

**1.4** How might ED boarding differentially impact certain residents or groups of learners?
*Probe*: In other words, do you think boarding has disparate impacts on certain groups of residents because of their identity? For example, have you noticed that residents of a certain race, gender, socioeconomic status growing up, other languages spoken etc. experience the boarding crisis differently due to different expectations placed on them?

- Is there any aspect of your identity that has [informed, altered, impacted] your experience working in an emergency room with patients who are boarding? When teaching residents how to practice emergency medicine in an environment with boarding?
- How might you see this as being different from how other educators (i.e., other attendings) have experienced it?

**1.5** When thinking about what it means to teach emergency medicine in an environment with high levels of boarding, can you tell me more about the relationship of learners to the patient populations they are serving?

**SECTION 2: Adaptations & Teaching Practices**
Now, let’s shift to how you, your fellow attendings, and your residents have adapted to the situations you’ve described.

**2.1** Tell me about a workaround, strategy, or adaptation—formal or informal—you or your team have developed to continue resident education when you felt there were more patients boarded than you could comfortably manage, or more generally, during times of increased boarding? How did it feel to experience the “workaround,” and how sustainable does it feel?
*Probe:* Which strategies have been most effective? Least effective?

**2.2** What does your role as “educator” look like during times of high boarding, and what kinds of responsibilities do you give residents? Is this different from times when there is little to no boarding?

**2.3** Do you feel like your expectations for resident responsibilities have shifted because of boarding? If yes, how?
*Probe:* Have you found residents doing more case management or inpatient-style work? If yes, how do you feel like they are prepared to do this work? Do you feel like it enhances or detracts from their education? How so? Have you had to shift what you teach to prepare residents for these roles? *How do you weigh those tasks against educational value?*

**2.4** How do you think the conditions you’ve described might influence your own practice as an educator—or your practice of emergency medicine more broadly?

**SECTION 3: Closing & Reflections**
We have touched on some of the impacts of boarding—how you have experienced them and how you have felt in a medical environment that has patients boarding. Lastly, I’d like to explore some final reflections.

**3.1** Do you feel that what you’re able to offer patients—and learners—is in line with the values that brought you to emergency medicine?

**3.2** How has your outlook on your career changed over the past few years in response to these systemic conditions?

**3.3** What suggestions do you have for improving resident education and/ or resident well-being in the setting of chronic boarding? What changes would you like to see to better support resident education and faculty well-being in the context of chronic ED boarding?

**3.4** Is there anything else you’d like to share about your experience with boarding that we haven’t covered?

**EMERGENCY DEPARTMENT BOARDING PROJECT**

Interview Guide for Medical Residents

**SECTIONS:**
Introduction (4)
Clinical Education & Skill Development (5)
Adaptations & Teaching Practices (4)
Closing & Reflections (2)

[Complete verbal consent script and obtain verbal consent before proceeding.]

[Acknowledge relevant demographic information already collected in pre-interview communications.]

**Research Question**
*How has emergency department boarding reshaped clinical education, bedside teaching, and skill development in Graduate Medical Education for Emergency Medicine, and how have residents and attending physicians adapted to the challenges and opportunities posed by the boarding crisis?*

# **INTRODUCTION: ED Boarding** Thank you for participating in this interview. This study seeks to understand how emergency department boarding impacts resident education, patient care, and physician well-being. Your insights will help us evaluate current conditions in the Emergency Department and identify both challenges caused by ED boarding and potential improvements to ED workflows and resident education.

**0.1** Can you describe to me what a typical shift of an emergency physician looks like?

**0.2** Overall, how would you describe the learning environment at each of the sites you have rotated at?

**0.3** As you may know, boarding refers to holding patients in the ED after they have been admitted or transferred to the hospital but are unable to be moved to an inpatient bed or other designated area due to lack of availability. At which sites have you noticed this occurring, if any? How did working in under these conditions feel?

**0.4** Think of a time when you were caring for a patient during a time of ED boarding. What was this experience like for you?

**SECTION 1: Clinical Education & Skill Development**
Next, I’d like to pivot to the impact of boarding on residency education. I am going to show you a graph representing the number of people boarded in the ED, as a reminder of when boarding may have been worse. [Share screen to present a chart ED boarding levels by month].

**1.1** Reflecting on this graph or your last experience with boarding in the ED, how might you describe the impact of boarding on your education?
*Probe:* Have you noticed any changes in the types or frequency of bedside teaching due to boarding? Can you give me an example of what this has looked like on a shift? What are your interactions with attendings like? What do educational opportunities look like? Are there specific aspects—procedural opportunities, patient volume, or diversity of cases—that have changed?

**1.2** The ACGME has developed milestones that residents are evaluated on biannually to track their progress and skill development.

[Review list of 6 milestone buckets with participant: patient care, medical knowledge, systems-based practice, practice-based learning and improvement, professionalism, interpersonal communication.]

- Think back to a time when more patients were boarded than usual. How do you feel like your progress on meeting these milestones was impacted during this time?
- During this time, how effectively are you able to demonstrate your competency or progression through these milestones to supervisors?
  - *Probe*: Do you think the additional tasks required during times of boarding (when light or heavy) impede or facilitate opportunities for supervisors to evaluate your progress with these milestones and observe your growth? Their ability to give feedback on your performance?
- Which milestones are most affected?

**1.3** How do you think boarding has affected your confidence or readiness in delivering emergency care (e.g., resuscitations, procedures, decision-making under pressure)?

**1.4** How might ED boarding differentially impact certain residents or groups of learners (including/ not including self)?
*Probe*: In other words, do you think boarding has disparate impacts on certain groups of residents because of their identity? For example, do residents of a certain race, gender, socioeconomic status growing up, other languages spoken etc. experience the boarding crisis differently due to different expectations placed on them?

- Is there any aspect of your identity that has [informed, altered, impacted] your experience working in an emergency room with patients who are boarding? How might you see this as being different from how other members of your cohort have experienced it?

**1.5** When thinking about what it means to learn emergency medicine in an environment with high levels of boarding, can you tell me more about the relationship you have to the patient populations you are serving, to the exposures you are receiving?
*Probe: For example, unhoused individuals, patients with limited English proficiency, patients with behavioral health needs, etc.*

**SECTION 2: Adaptations & Teaching Practices**
Now, let’s shift to how you, your attendings, and your program have adapted to the situations you’ve described.

**2.1** Tell me about a workaround, strategy, or adaptation—formal or informal—you or your team have developed to continue resident education when you felt there were more patients boarded than you could comfortably manage, or more generally, during times of boarding? How did it feel to experience the “workaround,” and how sustainable does it feel?

**2.2** Do you feel like expectations for resident responsibilities have shifted because of boarding? If yes, how?
*Probe:* Have you found yourself doing more case management or inpatient-style work? If yes, how do you feel like you are prepared to do this work? Do you feel like it enhances or detracts from developing skills relevant to your future practice as an Emergency Medicine physician? How so?

**2.3** How do you think your future practice of medicine might change because of the situations you just described?
*Probe:* Way of interacting with patients? View of the role of the physician? Your future career goals?

**2.4** In your experience, what has been the most effective strategy for dealing with boarding? Has this changed over time?

**SECTION 3: Closing & Reflections**
We have touched on some of the impacts of boarding—how you have experienced them and how you have felt in a medical environment that has patients boarding. Lastly, I’d like to explore some final reflections.

**3.1** What suggestions do you have for improving resident education and/ or resident well-being in the setting of chronic boarding? What would you like to see, or what would you like to see more of?

**3.2** Is there anything else you’d like to share about your experience with boarding that we haven’t covered?
